# A retrospective chart review of pirfenidone-treated patients in Sweden: the REPRIS study

**DOI:** 10.3402/ecrj.v3.32035

**Published:** 2016-07-18

**Authors:** Carl Magnus Sköld, Christer Janson, Åsa Klackenberg Elf, Marie Fiaschi, Kerstin Wiklund, Hans Lennart Persson

**Affiliations:** 1 Department of Medicine Solna, Karolinska Institutet, Lung-Allergy Clinic, Karolinska University Hospital Solna, Stockholm, Sweden; 2 Department of Medical Sciences: Respiratory, Allergy and Sleep Research, Uppsala University, Uppsala, Sweden; 3 InterMune Nordics AB, Stockholm, Sweden; 4 Roche AB, Stockholm, Sweden; 5 PCG Clinical Services, Uppsala, Sweden; 6 Division of Pulmonary Medicine, Department of Medical and Health Sciences, Faculty of Health Sciences, Linkö ping University, Linkö ping, Sweden

**Keywords:** idiopathic pulmonary fibrosis, pirfenidone, forced vital capacity, comorbidities

## Abstract

**Background:**

Idiopathic pulmonary fibrosis (IPF) is a chronic, progressive lung disease that usually results in respiratory failure and death. Pirfenidone was approved as the first licensed therapy for IPF in Europe based on phase III trials where patients with a forced vital capacity (FVC) >50% of predicted were included. The aim of this study was to characterise patients treated with pirfenidone in Swedish clinical practice and to describe the adherence to the reimbursement restriction since reimbursement was only applied for patients with FVC below 80% of predicted.

**Methods:**

This was a retrospective, observational chart review of IPF patients treated with pirfenidone from three Swedish university clinics. Patients initiated on treatment during the period 28 June 2012 to 20 November 2014 were included. Data on patient characteristics, basis of diagnosis, treatment duration, quality of life, and adverse drug reactions (ADRs) were collected from medical charts.

**Results:**

Forty-four patients were screened and 33 were included in the study. The mean treatment duration from start of pirfenidone until discontinuation or end of study was 38 weeks. At the initiation of pirfenidone treatment, FVC was 62.7% (12.1) [mean (SD)], diffusion capacity (DLco) was 45.1% (13.8) of predicted, and the ratio of forced expiratory volume on 1 sec (FEV1) to FVC was 0.78 (0.1). The percentage of patients with an FVC between 50 and 80% was 87%. Ten of the patients had ADRs including gastrointestinal and skin-related events, cough and signs of impaired hepatic function, but this led to treatment discontinuation in only two patients.

**Conclusion:**

Data from this chart review showed that adherence to the Swedish reimbursement restriction was followed in the majority of patients during the study period. At the start of pirfenidone treatment, lung function, measured as FVC, was lower in the present cohort of Swedish IPF patients compared with other registry and real-life data. About a third of the patients had ADRs, but discontinuation of the treatment because of ADRs was relatively uncommon.

Idiopathic pulmonary fibrosis (IPF) is an interstitial lung disease of unknown aetiology, which is characterised by remodelling and fibrosis of the lungs. This leads to a progressive loss of lung function over time, ultimately ending in respiratory failure and death. The median survival time after diagnosis is reported to be 2–5 years (1–4). IPF is generally diagnosed between 40 and 80 years of age with a prevalence peak at 65–79 years (4–6). The incidence is estimated at 4.6–8.8/100,000 per year, and the prevalence is estimated at 14–28/100,000 (5–9). The assumption is that, with a population of 9.9 million, approximately 1,500 individuals exist with IPF in Sweden, but the exact prevalence of the disease is unknown.

On 28 February 2011, pirfenidone (Esbriet^®^) was approved by the European Medicines Agency (EMA) for the treatment of adults with mild-to-moderate IPF (10). The EMA approval of pirfenidone was based on a pooled analysis of two pivotal phase III studies, CAPACITY 004 and CAPACITY 006, showing benefits on absolute change in per cent predicted forced vital capacity (FVC) (primary endpoint) and 6-min walking test (6MWT) after 72 weeks compared with placebo (11). Patients included in the pivotal trials had a per cent predicted FVC ≥50%. The approval of the drug was also supported by two earlier Japanese studies (12, 13).

After the approval in Europe, the results from a phase III study, ASCEND, as well as from an on-going open-label, extension of the CAPACITY studies, showed that pirfenidone treatment, as compared with placebo, reduced disease progression, as measured by changes in FVC and 6-min walking distance, and increased progression-free survival in patients with IPF (14, 15). In a pre-specified analysis from the ASCEND and the CAPACITY studies of the pooled population of all 1,247 included patients, it was shown that pirfenidone reduced the risk of death at 1 year by 48% compared with placebo (15).

As of 28 June 2012, the Swedish Dental and Pharmaceutical Benefits Agency (TLV) decided to include pirfenidone in the reimbursement system only for patients with a predicted FVC below 80% (16). The aim of the retrospective chart review was to characterise patients with pirfenidone in Swedish clinical practice and to describe the adherence to reimbursement imposed by TLV at the time of introduction.

## Methods

### Study design

The descriptive observational study, REtrospective PiRfenidone use In Sweden, REPRIS, was based on a retrospective review of medical charts (department clinical databases and hospital patient medical records) for patients treated with pirfenidone, at three Swedish university hospitals. The study has been assessed by the ethics committee as a quality assurance project. Patients at the participating clinics were included if they had started treatment with pirfenidone during the period 28 June 2012 to 20 November 2014 and were not participating in an interventional clinical trial or had previously participated in a named patient programme.

### Data collection

Data were retrospectively collected from medical charts. Information on the following variables was collected: patients’ referral pattern, age, sex, body height and weight, body mass index (BMI), smoking history, IPF diagnosis, comorbidities, pirfenidone treatment, use of IPF-related comedications, occurrence of adverse drug reactions (ADRs) related to pirfenidone treatment and IPF-related hospitalisations, reason for permanent discontinuation of pirfenidone treatment, and quality of life (QoL). In addition, FVC, the ratio of forced expiratory volume in one second (FEV1) to FVC, diffusion capacity (DL_CO_), and 6-min walking distance (6MWD) were also calculated.

### Statistics

The study was descriptive, and no hypothesis testing was performed; therefore, no sample size calculation was made. Statistical analyses were performed by PCG Clinical Services AB according to a predefined statistical analysis plan (SAP). All statistical analyses were performed using SAS^®^ (Version 9.4, SAS Institute, Inc., Cary, NC, USA), and continuous data were summarised using descriptive statistics and reported with the mean and standard deviation (SD), median, and minimum and maximum, as appropriate. For patients with data from more than one measurement at each time point (e.g. results from more than one pulmonary function test after treatment start), the mean of these values was calculated. Categorical data were summarised as frequency (*n*) and percentage (%).

## Results

### Patient characteristics

In total, 44 patients were screened and 33 were enrolled into the study (Table 1). The reasons for screening failure were pirfenidone treatment initiated prior to 28 June 2012 (*n=*3), participation in a clinical trial (*n=*4), or patient choice (*n=*4). The majority of patients had been referred to the university hospital clinic from a general practitioner (48.5%), from a private practitioner (pulmonary specialist, internal medicine, or cardiologist) (27.3%), or from other healthcare providers (24.2%) within the county. No patient had been referred from outside the county.

**Table 1:** Patient characteristics, diagnostic procedures, and comorbidities of enrolled patients

	Unit	Mean (±SD)
Age at start of treatment	Years	67.9 (9.8)
Min, max	Years	38.0, 87.0
Sex		
Male	*n*	25 (75.8%)
Female	*n*	8 (24.2%)
Smoking history		
Never	*n*	9 (27.3%)
Past	*n*	23 (69.7%)
Current	*n*	1 (3.0%)
BMI	kg/m^2^	26.4 (4.5)
Min, max	kg/m^2^	20.1, 38.0
Age at diagnosis	Years	66.6 (9.6)
Symptoms		
Dyspnoea	*n*	28 (84.8%)
Cough	*n*	28 (84.8%)
Reduced exercise tolerance	*n*	25 (75.8%)
Other	*n*	1 (3.0%)
IPF diagnosis based on		
HRCT	*n*	32 (97.0%)
Surgical lung biopsy	*n*	7 (21.2%)
MDT	*n*	19 (57.6%)
Other	*n*	5 (15.2%)
Presence of pulmonary and other comorbidities at start of treatment	*n*	29 (87.9%)
Emphysema appearance on HRCT	*n*	2 (6.1%)
COPD	*n*	1 (3.0%)
Lung cancer	*n*	0
Pulmonary hypertension	*n*	2 (6.1%)
Gastro-oesophageal reflux	*n*	14 (42.4%)
Diabetes mellitus	*n*	4 (12.1%)
Coronary heart disease	*n*	9 (27.3%)
Hypertension	*n*	12 (36.4%)
Other	*n*	22 (66.7%)

Data are presented as %.

Other apply to spirometry, bronchoscopy or bronco alveolar lavage (BAL).

BMI, body mass index; SD, standard deviation; HRCT, high-resolution computed tomography; MDT, multidisciplinary team.

The IPF diagnosis was based on high-resolution computed tomography (HRCT) in 32 out of 33 patients. However, documentation of HRCT was not available for one patient diagnosed outside the participating university clinic. One-fifth of the patients had a surgical lung biopsy. For over half of the patients (56.7%), diagnosis was based on discussion at a multidisciplinary conference (MDC). The age at diagnosis was 66.6 (9.6) (mean, SD) years and 67.9 (9.8) years at start of pirfenidone treatment. The majority of patients were men (76%), and most patients had a history of smoking (73%) (Table 1). Gastro-oesophageal reflux disease was the most common comorbidity (42%), followed by arterial hypertension (36%) and coronary heart disease (27%).

### Lung function

At the start of pirfenidone treatment, FVC was 62.7% (12.1) [mean (SD)], haemoglobin corrected DLco was 45.1% (13.8) of predicted, and the FEV1/FVC ratio was 0.78 (0.1) (Table 2). The percentage of patients with a FVC between 50 and 80% was 87%. Two of the patients had been initiated on treatment with FVC <50% and two with FVC >80% (Table 2). After the start of the treatment, until the end of study or discontinuation, results on FVC were missing in about half, and DL_CO_ in about 60% of the patients. Information on the FEV1/FVC ratio was captured at treatment start only. Six MWD was missing in the medical charts for the majority of the patients.

**Table 2:** Pulmonary function tests at start of pirfenidone treatment

	Unit	Mean (±SD)
Percent predicted FVC	%	62.7 (12.1)
Min, max	%	30.0, 91.0
Categorised percent predicted FVC		
< 50	*n*	2 (6.7%)
50–80	*n*	26 (86.7%)
> 80	*n*	2 (6.7%)
FEV_1_/FVC		0.78 (0.1)
Min, max		0.4, 1.0
Percent predicted DLco	%	45.1 (13.8)
Min, max	%	14.0, 65.0

DL_CO_, diffusing capacity for carbon monoxide (haemoglobin corrected); FEV_1_, forced expiratory volume in 1 sec; FVC, forced vital capacity; SD, standard deviation.

### Other medications and side effects

At the start of pirfenidone treatment, the most common other medication was *N*-acetylcysteine, followed by corticosteroids and oxygen therapy (Table 3). Proton-pump inhibitors were used by about 42% of patients prior to pirfenidone treatment (Table 3).

**Table 3:** Other medications prior to and at start of pirfenidone treatment

	*n* (%)
IPF-related treatment	
Immediately before treatment start	
Corticosteroids	6 (18.2%)
*N*-acetylcysteine	13 (39.4%)
Oxygen therapy	4 (12.1%)
Immediately after treatment start	
Corticosteroids	7 (21.2%)
*N*-acetylcysteine	10 (30.3%)
Oxygen therapy	5 (15.2%)
Other	1 (3.0%)
Gastrointestinal treatment	
Immediately before treatment start	
Proton-pump inhibitors	14 (42.4%)
Immediately after treatment start	
Proton-pump inhibitors	14 (42.4%)
Other	1 (3.0%)

Ten patients had at least one ADR related to pirfenidone treatment (Table 4). The ADRs included gastrointestinal and skin-related events, cough and an increase in liver enzymes and lead to pirfenidone dose adjustment, temporary or permanent stop of treatment, or use of other medications. During the study period, eight patients discontinued treatment with pirfenidone, while 23 patients still used the drug at the end of the study. All patients who discontinued treatment were men; the reasons for discontinuation were death (*n=*4), ADR related to pirfenidone (*n*=2), patient decision (*n=*1), or other (*n=*1). No patient discontinued due to disease progression, exacerbations, or lung transplantation. No lung function tests were performed when the treatment was stopped; thus, no results for this time point are available.

**Table 4:** Adverse drug reactions related to pirfenidone according to the treating physician and actions taken

	Patient, *n* (%)	Event, *n*
Adverse drug reactions	10 (30.3%)^a^	12
Gastrointestinal disorders	4	4
Respiratory, thoracic, and mediastinal disorder	2	2
Psychiatric disorders/metabolism and nutrition disorder	1	2
Skin and subcutaneous tissue disorders	2	2
Hepatobiliary disorder	1	1
General disorders and administration site conditions	1	1
Action taken		
None	1 (3.0%)	1
Hospitalisation	0	0
Pirfenidone dose changes	4 (12.1%)	5
Temporary stop of treatment with pirfenidone	3 (9.1%)	3
Permanent stop of treatment with pirfenidone	2 (6.1%)	2
Other medications	1 (3.0%)	1

a Patients may have experienced ≥1 ADR.

### Duration of treatment

The mean duration from IPF diagnosis until start of pirfenidone treatment was 55 (110) weeks, and the mean treatment duration from start until discontinuation or end of study was 38 (29) weeks. The dosing titration scheme of pirfenidone followed the summary of product characteristics (SmPC), and all patients were treated with three capsules three times daily at discontinuation or at the end of study (whichever applied).

## Discussion

The main finding in the present study was that adherence to the Swedish reimbursement restriction for pirfenidone was followed in the majority of patients. About one-third of the patients had ADRs but discontinuation of the treatment because of ADRs was relatively uncommon. Based on openly available data from the Swedish Prescribed Drug Register, 106 patients were prescribed pirfenidone during the year 2013 (17). Thus, a crude estimate is that approximately one-third of all patients treated with pirfenidone in Sweden were included in this study. Among the 106 patients who were prescribed pirfenidone in Sweden during the year 2013, 48% were aged 70 or older and 73% were men. This is similar to the population in the current study, in which the mean age was 68 years and 76% were men, and the data are also in line with the CAPACITY and ASCEND studies (11, 15).

In-line with international and national guidelines, the majority of patients were discussed within MDCs (1, 18). In the vast majority of patients, diagnosis was based on HRCT, and in 20% of patients a surgical lung biopsy was performed. This was in the same range reported both in a retrospective study from one of the included hospitals (19) and in the Swedish IPF Registry (20, 21).

In this retrospective analysis, patients were initiated on treatment with a lower FVC, 63% compared with other real-life data, in which ranges of 61–77% have been reported (22). On the other hand, the DLco was quite similar, 45% compared with 40–56% from other materials (22). As a comparison, in the Swedish IPF registry (20, 21), the mean FVC at diagnosis was 72.3%. The reason for the lower FVC at the start of treatment may be that patients were diagnosed at a later stage in Sweden or, more likely, that treatment was not initiated at the time of diagnosis. Another possible explanation is that the drug was only reimbursed for IPF patients with FVC <80%. In the Swedish IPF guidelines, published in 2012 (18), it is stated that asymptomatic IPF patients with normal lung function should have evidence of disease progression before treatment initiation, which may also have influenced respiratory physicians to wait.

The percentage of patients with a predicted FVC within 50–80% was 87% at the start of the treatment. Thus, the TLV restriction was followed in the majority of patients. Notably, since May 2015, the TLV reimbursement restriction has been removed.

Besides objective measurements of disease severity and progress, for example FVC, it is important to follow-up on patients’ QoL. Unfortunately, analysis of QoL assessments could not be performed due to too few observations. However, a translated QoL questionnaire (King's Brief Interstitial Lung Disease, K-BILD) has recently been developed and is being implemented in routine clinical practice in Sweden (20, 21).

The interest and knowledge within the field of IPF field is rapidly evolving, and it is of considerable interest to follow-up changes in clinical practice through registries, as the recently started Swedish IPF registry. This registry enrols data on IPF patients in Sweden to evaluate treatments and encourage collaboration between clinics with the overarching goal of improving patient care.

Data sets based on retrospective medical chart reviews mimic reality but have limitations. The medical records are used for clinical practice and not primarily for research purposes. As a consequence, data may be missing and, additionally, a small number of participating sites and few patients may limit the extrapolation of the result to the whole population.

## Conclusions

The findings from this Swedish retrospective chart review of patients initiating treatment with pirfenidone in clinical practice showed that the absolute majority of patients initiated on pirfenidone treatment adhered to reimbursement restrictions imposed by TLV at the time of evaluation. The knowledge within the IPF community is rapidly evolving, and the implementation of registries into routine clinical practice will enable the long-term follow-up of IPF patients.
